# A Pocket Epitome of the British Pharmacopœia

**Published:** 1892-03

**Authors:** 


*A Pocket Epitome of the British Pharmacopœia.* By RUSSELL COOMBE, M.A., F.R.C.S. Pp. vi., 90. London: Baillière, Tindall and Cox. 1891.

The title of this little book, which can easily be carried in the waistcoat pocket, indicates the nature of its contents. The epitome seems to be well and accurately done, is arranged in a manner suitable for ready reference, and the type is clear.

